# Patterns of ‘balancing between hope and despair’ in the diagnostic phase: a grounded theory study of patients on a gastroenterology ward

**DOI:** 10.1111/j.1365-2648.2007.04523.x

**Published:** 2008-04

**Authors:** Tove Giske, Barbara Artinian

**Affiliations:** Tove Giske MNSc RN Associate Professor, Research Fellow Bergen Deaconess University CollegeBergen, Norway; and Haukeland University HospitalBergen, Norway; Barbara Artinian PhD RN Professor Emeritus Azusa Pacific University School of NursingAzusa, California, USA

**Keywords:** despair, diagnostic phase, gastroenterology, grounded theory, hope, interviews, nursing, patients experiences, theory development

## Abstract

**Title:**

**Patterns of ‘balancing between hope and despair' in the diagnostic phase: a grounded theory study of patients on a gastroenterology ward**

**Aim:**

The aim of the study was to learn how patients going through the diagnostic phase experienced and handled their situation.

**Background:**

Many studies report about the stressful diagnostic phase; however, none has presented a conceptual theory where the concepts are sufficiently related to each other. The Theory of Preparative Waiting has previously been published as a descriptive grounded theory and describes the experience of a group of gastroenterology patients going through the diagnostic phase.

**Method:**

A classical grounded theory design was used, with data derived from 18 in-depth interviews with 15 patients in a gastroenterology ward at a Norwegian University Hospital. Interviews were conducted during 2002–2003.

**Findings:**

Participants' main concern was found to be how they could prepare themselves for the concluding interview and life after diagnosis. The theoretical code of ‘balancing’ had four patterns; controlling pain, rational awaiting, denial, and accepting. These patterns of ‘balancing’ guided how participants used the categories of ‘Preparative Waiting Theory’‘seeking and giving information’, ‘interpreting clues’, ‘handling existential threats’ and ‘seeking respite’. Patterns were strategies, so one person could use more than one pattern.

**Conclusion:**

The diagnostic phase was a difficult time for the participants and the ‘Preparative Waiting Theory’ can assist nurses in assessing how patients prepare themselves differently for getting a diagnosis. All patients would find it helpful to be followed up by a designated contact person at the ward; however, patients using mostly the patterns of controlling pain and denial would benefit most from such support.

**What is already known about this topic**

- Theoretical coding is the least understood part of grounded theory analysis.
- The ambiguity of the diagnostic phase causes uncertainty and is experienced as the most stressful part of the illness trajectory for patients.
- The informational needs and different ways of dealing with the emotional challenge of waiting in the diagnostic phase are widely acknowledged.

**What this paper adds**

- A conceptual grounded theory where the processes of preparing for a diagnosis are related to each other through the theoretical code of ‘balancing’.
- Articulation of how the four ‘balancing’ patterns of controlling pain, rational awaiting, denial and accepting explain how patients prepare differently for getting a diagnosis.
- A model which could be used in developing the nursing role for patients in the diagnostic phase.

## Introduction

Most patients admitted to hospitals for diagnostic investigations go through a challenging time of waiting. The entire situation is filled with waiting; waiting for investigations and tests, preparing for and undergoing them, and finally waiting for results. They do not know what kind of disease they may have, possible life changes that are in store, or treatment it will possibly require. The time of waiting for a diagnosis is reported to be the most stressful time of the illness experience (Mishel 1988, 1997, Benedict *et al.* 1994, Fridfinnsdottir 1997, Poole 1997, Poole & Lyne 2000, Lebel *et al.* 2003, Neville 2003).

In this paper, we report the full analysis and development of the Theory of Preparative Waiting, which has previously been published as a descriptive grounded theory (Giske & Gjengedal 2007). The theory describes the experience of a group of gastroenterology patients going through the diagnostic phase. This paper shows how the theoretical code of ‘balancing between hope and despair’ integrates the theory and how the theory is related to other research.

After developing our substantive ‘Preparative Waiting Theory’ (PWT), we did a literature research to compare our findings with other research in the area. We found no papers reporting from the same patient group, or with as open an outcome of the investigation as in our group, which led us to widen our search for relevant literature. We found reports of investigations of breast anormalities (Benedict *et al.* 1994, Heskestad & Tjemsland 1996, Fridfinnsdottir 1997, Ambler *et al.* 1999, Thorne *et al.* 1999, Poole & Lyne 2000, Woodward & Webb 2001, Drageset & Lindstrøm 2003, 2005, Lebel *et al.* 2003, Logan *et al.* 2006). There were also studies of younger patients waiting for diagnosis in an acute ward (Sørlie *et al.* 2006), studies related to waiting for liver transplantation (Jonsèn *et al.* 2000), waiting for colon surgery (Moene *et al.* 2006), waiting for diagnostic tests in chronic back pain (Rhodes *et al.* 2002), and waiting for surgery of knees and hips (Sjöling *et al.* 2005).

**Figure 1:**
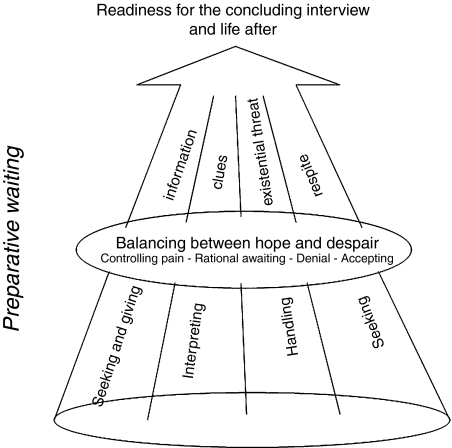
Model of Preparative Waiting Theory.

Our substantive grounded theory (GT) of PWT was a process of balancing between ‘seeking and giving information’, ‘interpreting clues’, ‘handling existential threats’ and ‘seeking respite’, as illustrated in Figure 1. By comparing these research reports with our PWT, we found that most studies dealt with the emotional part of waiting and the need for information. To a lesser extent they dealt with existential aspects of waiting and how patients interpreted clues and sought respite. Just one study (Heskestad & Tjemsland 1996) described all the aspects we identified in our study; however, the researchers did not organize them into higher level concepts nor relate them to each other.

In GT, researchers search for theoretical codes that can conceptualize and integrate a substantive theory (Glaser 1978, 2005). In our study ‘balancing’ emerged as such a theoretical code. In a study by Thulesius *et al.* (2003) the theoretical code of ‘balancing’ described how health care professionals balanced the needs of end-of-life patients with available resources. They suggested that ‘balancing’ is a fundamental process operating in all types of health care (p. 1371). Similar findings were reported in nursing literature. Irurita and Williams (2001) found that patients and nurses in acute care settings were in a reciprocal process of balancing and compromising to preserve the integrity of one's self and of others. In a GT study of AIDS/HIV patients, Kylmä*et al.* (2001) reported that patients balanced between hope, despair, and hopelessness in everyday life.

Against this background, this paper presents and discusses PWT from the perspective of how the different patterns we identified of ‘balancing between hope and despair’ affected the ways participants sought information, interpreted clues, handled existential threat, and sought respite. This use of the theoretical coding of ‘balancing’ moved our analysis from the descriptive GT previously published (Giske & Gjengedal 2007) to an integrated theory.

## The study

### Aim

The aim of the study was to learn how patients going through the diagnostic phase experienced and handled their situation.

### Method

Classical GT was chosen as the method (Glaser 1978, 1998, 2005). GT is well suited for studying complex and hidden processes (Morse 2001). Full details of study methods appear in Giske and Artinian (2007) and a brief outline follows here. In GT, data collection and analyses are performed concurrently. The data are first coded openly, later selectively according to the core concept, and finally theoretically. The purpose of theoretical coding is to relate the substantive codes to each other in such a way that the theory fits data, is relevant by letting the processes in the field emerge, and works to explain what is going on in the area studied (Glaser 1978).

### Participants

Eight women and seven men aged from 35 to 84 and admitted to a gastroenterology ward participated in the study. The age of 35 years and older was chosen because at this age most people are settled in the most important roles in their lives. When interviewed, their health problems had lasted from 1 day to 9 years. Participants were chosen in accordance with the development of the theory (theoretical sampling).

### Data collection and analysis

Data collection (in-depth interviews) and analysis were performed concurrently in 2002–2003. The 18 tape-recorded and transcribed interviews (three patients were interviewed twice) were analysed by open coding, moving on to selective coding when the participants’ main concern was found to be ‘how can patients prepare themselves for the concluding interview and future life’, and the core category ‘preparative waiting’ became clear. Memos were continuously written to aid the process of theoretical coding, which fitted the concepts to each other to constitute a dense and parsimonious GT.

In the analysing process, we developed many concepts. Two of the most distinct were ‘vulnerable dependency on staff and system’ and ‘balance between hope and despair’. The first contained participants’ experiences and strategies related to staff and being in the hospital environment. This category was eliminated because it described the condition of being a patient and had to be a condition for all categories. The second concept was early seen to be more guiding for the process than the other concept and we also identified four different patterns of how ‘balancing’ was performed. As data analysis continued, ‘balancing’ emerged as the theoretical code that integrated all the other categories.

Theoretical codes are abstract models allowing the researcher to talk about substantive categories and properties while thinking theoretically (Glaser 2005, p. 3). Theoretical codes are flexible, and more than one may fit the same data. Glaser (1978, 1998, 2005) gives many examples of theoretical codes, one of which is ‘balancing’. ‘Balancing is handling many variables at once to start an action, keep an action going or achieve a resolution … Balancing is an abstract model that can be seen substantively or used as a substantive category’ (Glaser 2005, p. 29). Theoretical codes are ‘hard to understand in the beginning of research by the novice GT researcher’ (p.1). Therefore they are often ignored, left implicit, or just missed. A theoretical code is not necessary for a GT, but ‘a GT is best when they are used’ because ‘a GT will appear more plausible, more relevant and more enhanced when integrated and modelled by an emergent theoretical code’ (p. 14). This became significant in our study. When the theoretical code of ‘balancing’ emerged, it served to integrate the entire participants’ experience. It also raised the PWT to a higher level, so that we could state hypotheses about relationships between patterns and categories.

### Ethical considerations

The study was approved by the Region's Medical Ethics Committee. Participants were recruited to the study in collaboration with nurses on the ward, who prepared patients by giving oral and written information. The interviews started when participants had signed the consent form.

## Findings

### ‘Balancing’: the theoretical code integrating PWT

The theoretical code of ‘balancing’ shows how participants in the diagnostic phase resolved their main concern by ‘seeking and giving information’, ‘interpreting clues’, ‘handling existential threat’ and ‘seeking respite’. ‘Balancing’ was an intrinsic activity where participants moved between hope and despair. All participants had emotional boundaries concerning how deep into despair they could go before losing control, or how focused on hope they could be without losing track of reality. If they let despair and their worst fear become too strong, controlling and hiding how painful their situation was and how weak they actually felt, became difficult. This moving between hope and despair influenced for example how they sought and processed information. The more hopeful they were, the more realistically they could appraise and process their situation but when they moved towards despair, they withdrew and were less able to process their situation. Different patterns of ‘balancing between hope and despair’ emerged from the data. Patterns are behaviours patients engage in; they are strategies rather than labels, and so a person can use more than one pattern (Glaser 2001). Four patterns were identified.

*Controlling pain*: participants wondered what the truth might be about their situation, and they felt the pain of uncertainty about the future in large measure. To protect themselves, they controlled their thoughts related to future prospects: ‘I am nervous and tense, but I do not think my thoughts all the way through and finish’. The pattern of *rational awaiting* was evident when a person focused on facts instead of hypothetical outcomes. Troublesome thoughts and feelings were not allowed to emerge as long as patients lacked accurate knowledge about their status. One man put it this way: ‘You cannot be anxious about things you do not know in the process, you must wait until you have a result. I keep it at a level that I can handle’. When the pattern of *denial* was recognized, participants focused on recovering from acute symptoms rather than brooding over possible underlying problems: ‘I have been ill for many weeks, and now I recover so fast. I have not been thinking so much about how ill I am. What causes the enlarged liver I do not know, and I do not care so much about it either’. The fourth pattern, *accepting*, was identified when participants had the confidence to openly seek information about what was at stake. The pain caused by fear of the future was combined with peace stemming from the belief that they would be able to cope. As illustrated in this quote; age, experience in life, and ability to reflect on former experiences were important conditions in this strategy: ‘I hope for an outcome, but if not, I believe I will have no problem with facing it. I have some life experiences that will help me’.

### Seeking and giving information

The first concept of PWT was related to how participants sought and gave information. They sought information related to the content of their stay such as structure of the day, and how to prepare physically and mentally for investigations and their results. Such information gave them some control and possibilities of judging their situation. Participants saw it as the physicians' and nurses' responsibility to give the correct information to them in such a way that it was not misinterpreted. They also expressed a wish to have one main contact person in the medical team. The longer the patient had been waiting for a diagnosis, the more important it was that everything was performed correctly to ensure accuracy of results. Participants also wanted to give the healthcare team information about how they experienced their symptoms so that physicians could be able to make the right judgment about their situation.

Participants themselves could be an obstacle to information exchange because worry, fear and their physical state limited their ability to process information. They sometimes heard and understood only parts of information given to them at doctors rounds. Some were afraid to ask or forgot what they had planned to ask the physician during ward rounds.

The more participants felt on an even keel, the more they were able to process information and the better prepared they became. Information was best accepted when it was given in accordance with patterns of their balancing work. Those using the pattern of controlling pain neither wanted too much information nor wanted to be involved in discussions about all outcomes with regard to care. One put it this way: ‘It is OK that they are worried [physicians] and wonder what this can be. For me it is better to get to know when they have something more specific to tell’. Building trusting relationships with staff became a resource to lean on during their painful uncertainty. In contrast, poorly prepared ward rounds and new staff asking the same questions repeatedly drained them of hope and increased their despair.

Participants using the pattern of rational awaiting wanted information so that they could base their judgments on facts. Emotions related to different outcomes were kept on hold until they knew for sure what the outcome would be: ‘For me it is important to get a diagnosis, a serious one or a less serious one. I am concerned about the different alternatives, but I am waiting for the results to see how I can relate to it. I am a realist and this is how I am used to thinking’.

Denial was seen when participants ignored signs of danger and overlooked negative possibilities. Some saw serious questions asked by physicians as impolite: ‘I have this itch, you see. When I was admitted, the physician pointed out that I had a problem with alcoholism. But there can be other reasons for this, [such] as kidney stones or a blocked blood vessel. One should be careful and not destroy people's self-esteem’. With this pattern, the themes of an interview could change rapidly, jumping away from threatening topics only to return later and touch them briefly. Participants using the pattern of accepting sought information about their situation, and they were able to process it and relate to the emotions associated with the range of potential diagnostic outcomes.

### Interpreting clues

The second concept dealt with how patients interpreted clues. Participants remembered what had happened before they became ill, and they considered the changes in their body. As they had different levels of medical knowledge and experience with illness and diseases, they interpreted bodily symptoms such as bleeding from the rectum, feeling lethargic, having a raised temperature, pain, diarrhoea, enlarged liver, and weight loss differently. In addition to reflecting on their bodies, they paid attention to and considered how they were met in the hospital, what diagnostic interventions they went through, and what priority they were given. These internal considerations, together with interpretation of the interplay between themselves, staff and the organization, were properties of the category of ‘interpreting clues’ in our study.

The different patterns of ‘balancing’ were related to how participants evaluated their situation. Participants using the controlling pain pattern were very sensitive to all clues, because of the vulnerability of feeling the distressing uncertainty. For self-protection, they did not allow themselves to consider fully the range of outcomes. Those using the rational awaiting pattern interpreted as many comprehensible clues as possible to try to make sense of their situation. As emotions were put aside until the diagnosis and the prognosis were known, they hardly felt the emotional pain of considering the different scenarios: ‘They are reluctant to tell me something before they know, and this biopsy, that is what is decisive. It is obvious that it is considered to be a rather serious affair because it has been given top priority in this examination’. Those using the pattern of denial turned their attention mainly towards positive signs, and compared their own situation with others who manage to live well despite diseases. When the pattern of accepting was seen, clues were considered and compared with knowledge and former experiences. One man shared his attempts at trying to make sense of his situation: ‘I have an ulcer in my duodenum that will not heal, and it is bleeding, and I have this increasing pain. You were thinking of cancer…Today, the ultrasound showed a narrowing in the duodenum, and I am very tense about the result, if it is malignant or…’.

### Handling existential threat

The third concept was related to their experience of going through medical investigations and waiting for a diagnosis, which made participants consider possible outcomes of their illness. They were apprehensive about the future and what it might hold, questioning what might change for them and their loved ones. These concerns were intrinsic and often not expressed. Many outcomes were possible: it could be a serious and life-threatening disease leaving them with reduced lifespan, a chronic disease, a somewhat treatable problem easily fixed, or nothing at all. The possibility of more serious threats challenged them to reflect on what really gives meaning to life. Being admitted to hospital added seriousness to the situation, and the smells, sounds and sights of the hospital, staff and fellow patients made it harder to hide from these thoughts.

To be in the diagnostic phase meant to be kept on hold in life and, even though they became more aware of meaning, this was not the time for making changes in such basic matters as outlook on life. For those having a Christian faith, a variety of images of God were found, which again influenced their way of seeking God in this hour of need. The way they experienced the existential threat was closely linked with the different patterns of ‘balancing’, as stated in the hypothesis that the more painful they felt the situation, the greater they felt the existential threat.

The pattern of controlling pain was used to protect themselves from dwelling too much on what they feared could be a serious outcome. For those believing in God, praying was a way of handling their distress:

I believe in God and the faith has become important to me. I pray; most of us pray when we are in some kind of need, I think. I say: God help me now (she cries) – I cannot take anymore, now I need help.

When the pattern of rational awaiting was seen, the handling of uncertainty for the future was dealt with by not considering the consequences of different outcomes before they had more facts about the situation. Their attitude was that nothing had changed before they had been given a diagnosis: ‘I am a realist, and I think that will help me, because I am not the type of person that can stand lulling myself down in things. I am impatient and want to get on with life’. The nature of the denial pattern was to not to delve into threatening prognoses, which resulted in limited exploration as to what this could mean for the future. When acceptance was seen, participants discussed both the distress of an uncertain future and the trust related to the ability to handle their situation. This trust was built on former experiences of their own resources and available support of others:

There is a lot of uncertainty related to my future and what I can manage in relation to my family. He cries. This is very painful for me. I have learned that life is not easy, but I have managed to be the strong one in the family, and my previous experiences have taught me how to cope.

### Seeking respite

The fourth concept was ‘seeking respite’. This dealt with stepping out of the constant tension of uncertainty, as when participants worked on ‘balancing’ their hopes and despairs, making sense of their situation, handling worries for the future and seeking an alternative mental state where their thoughts were concerned with things other than waiting. Respite granted rest and renewed strength: it offered a mental escape where they could disconnect from the smouldering tension of uncertainty, and it helped to ‘recharge the batteries’, to prevent them from ‘exploding’ or ‘become crazy’, as participants expressed it.

Different conditions influenced the ability to find respite, such as where they slept – in the hospital, at the patients' hotel (nearby housing where patients could stay when health and the examination programme permitted), or at home. How obedient or undemanding they were also made a difference. Conditions such as pain or nausea and preparations for investigations such as fasting also made it harder for participants to disconnect. In the hospital, respite was found through reading, listening to music, walking in the garden and chatting with fellow patients.

Different patterns of balancing were also identified in ways of seeking respite. The more painfully they experienced their situation, the more they felt the need for respite. When the pattern of controlling pain was seen, respite was sought to find a break from the pain caused by uncertainty. Such rest gave renewed strength to endure new investigations and prolonged waiting. One woman put it this way:

When you can have leave and go home, I get more input from friends. I have the house and a dog – things that can occupy my thoughts. I need to recharge the batteries between the battles. When you are here, you are in bed and you see nurses, physicians and fellow patients, and then all the smells. You do not get the chance to disconnect as easily here, even though I try.

For those using the pattern of rational awaiting, respite helped to ‘kill’ time as they moved towards a conclusion. Respite was actively sought when denial was used; it offered a welcome break from bothersome threats in the back of their minds. The use of humour was tangible, and participants could keep themselves busy by following up on fellow patients' needs.

For those using the pattern of accepting, respite was also appreciated as it gave a welcome break from their work of dealing with uncertainty: ‘I am doing a lot of thinking while I am here. I read a book and listen to music to enter another world. I need to overcome a threshold to move away from what my head is full of’ (p. 11).

### Relationships between the categories

Table 1 summarizes how the theoretical code of ‘balancing’ integrated the categories of PWT, and shows how different participants prepared themselves differently for getting a diagnosis.

**Table 1:** Relationship between the different patterns of ‘balancing between hope and despair’ and the other concepts of Preparative Waiting Theory

Patterns of balancing between hope and despair	Seeking and giving information	Interpreting clues	Handling existential threat	Seeking respite
Controlling pain				
Pain of uncertainty feltto a great extent	Wanting accurateinformation, not toomuch into discussionabout differentpossibilities	Work on making senseof all possible clues.Do not go into theworst scenarios	Feel the existentialthreat to a great extent.Try to pull themselvesback from going toomuch into it	Seek respite as it gives awelcome break fromthe painful time ofwaiting that gives restand renewed strength
Rational awaiting				
Focused on facts and noton hypotheticaloutcomes	Focus on facts, not onhypothetical outcomes	Work on clues to makethe best estimate oftheir situation	Nothing has changed.The existential threat isput on hold untilknowledge andconsequences aboutthe situation areknown	Seek respite because ithelps killing the timewhile waiting for theconclusion of themedical examination
Denial				
Focused onimprovements andoverlooked signs ofdanger	Information is notactively sought.Recognize thatsomething is wrong,and accept the need toknow the result to beable to get help	Focus on improvementsand all positive signs inthemselves. Comparethemselves with otherswho got well or whomanaged to live wellwith disease	To a limited extentgo into this	Seek respite actively as itoffers a welcome restfrom the bothersomethreat of uncertainty
Accepting				
Uncertainty wasbearable as there wasconfidence in enoughresources available tocope with the situation	Seek and want accurateand adjustedinformation	Work on making senseof all clues. Considerformer experiences ofcoping with difficultlife situations andcompare them with theactual situation	Experience the situationas less a threat as theyfind some rest inknowing there areresources available forthem in relation tothemselves, tosignificant others, andto God	Need less respite as theemotional pain ofuncertainty is bearable

## Discussion

In discussing the findings, we must bear in mind that data from this study came from only one ethnic group and location. Understanding the theoretical code of ‘Balancing’ and how the patterns of controlling pain, rational awaiting, denial, and accepting relate the strategies of PWT to each other makes it easier to be sensitive to how different patients prepare themselves. But it will still be demanding for nurses to develop supportive interactions with patients. Patients’ processes of preparing are dynamic, moving between hope and despair, levels of awareness and more or less realistic appraisal of their situation (Giske & Gjengedal 2007). A person can also move between different patterns, even though there was a tendency to use one pattern more than the others. This wavering between hope and fear is explicitly discussed by Benedict *et al.* (1994) and Heskestad and Tjemsland (1996). Fridfinnsdottir (1997) identified three ways of coping which she called denial, stoic acceptance, and selective attention. The first two ways are comparable to our patterns of denial and rational awaiting, and the last is best compared with the strategy of seeking respite. The different patterns made participants become prepared to various degrees; participants using mostly the pattern of accepting would be better prepared than those using mostly denial. This is in accordance with Heskestad and Tjemsland (1996), Fridfinnsdottir (1997), Poole and Lyne (2000), Widerman (2004), and Drageset and Lindstrøm (2005).

Patients using mainly the pattern of controlling pain go through a very harsh time, experiencing a great deal of emotional pain. Building of trusting and caring relationships, and continuous contact with a few key people would offer these patients healthcare resources to lean on that would strengthen their hope and support them emotionally through the investigation process. Awareness of the existential pain these patients feel and their need to distance themselves from thinking about the worst scenarios should make staff aware of how they communicate potential possibilities along the process. To assist patients in reaching respite would be a way to help them gain a break from uncertainty-provoking anxiety. Such a break would give them renewed strength to endure the investigation process.

Patients mostly using the pattern of rational awaiting have limited conscious contact with emotions related to the uncertainty of their situation. They postpone emotional processing of their situation until they know the outcome. In relation to these patients, nurses need to consider to what degree they see it as helpful for patients to connect emotions to the cognitive preparation they go through. Newer theories of grieving after bereavement imply that it is possible to work thorough stressful situations without emotional catharsis (Stroebe *et al.* 2006, Guldin 2007). Accurate information is highly valued by these patients.

Patients using denial to a large extent tend to distort the judgment of their situation to protect themselves from the emotional threat of bad scenarios for the future. To the extent that nurses earn these patients' trust, they can strengthen patients' hope and thereby reduce the need for denial and other subconscious processes (Giske & Gjengedal 2007). By demonstrating competence, continuity, and care, nurses can assist patients in a more realistic appraisal of their situation and thus help them to prepare more accurately for the concluding interview in which they receive the diagnosis and for life afterwards.

Patients mainly using the pattern of accepting seem to be the ones who handle their situation best. These patients are able to appraise their situation realistically, trusting that there will be enough resources for them to cope with whatever the outcome might be. The situation of uncertainty and waiting is painful for these patients too, and a contact person, a well-coordinated investigation programme and continuity of physicians and nurses would make the situation more predictable for them and thereby ease their pain.

If we look at general implications of this study, one overall recommendation is to assign a knowledgeable and experienced nurse to each patient entering the hospital for diagnostic workups. Such a contact person could coordinate the investigation process and provide patients with accurate information and thereby reduce the pain of uncertainty. Information about hospital routines and details of procedures can improve patients' sense of control. Similar findings are reported by others (Ambler *et al.* 1999, Thorne *et al.* 1999, Woodward & Webb 2001, Drageset & Lindstrøm 2003, Lebel *et al.* 2003, Sjöling *et al.* 2005).

It is important for nurses and physicians to be aware of how patients feed clues into the process of making sense of their situation. Our participants tried to judge the seriousness of their symptoms by comparing their ongoing evaluation of the situation with knowledge and previous experiences in their own and others’ lives. We found just one study that reported how patients tried to make sense of their bodily symptoms when no objective proof could be found (Rhodes *et al.* 2002). Participants in our study also paid attention to non-verbal clues in staff behaviour and how staff appeared. Other studies report similar findings related to non-verbal signs from staff, such as facial expressions, gaze of the eye, tone of voice, and gestures (Heskestad & Tjemsland 1996, Thorne *et al.* 1999, Poole & Lyne 2000). Our participants also interpreted the investigations and the priority they were given, a feature also recognized by others (Thorne *et al.* 1999, Poole & Lyne 2000). These findings show that patients invest time and personal interest in reducing uncertainty related to their case, and in doing so they take in as much information as they are capable of processing.

‘Handling existential threat’ was one of the main concepts of ‘preparative waiting’. The more participants felt the pain of uncertainty, the more they consciously dealt with the existential threat. Not knowing their diagnosis and prognosis challenged them to consider what was important in life. All participants pondered over the image of God, even though some of them had a non-religious worldview. The same results are reported in other Norwegian studies (Heskestad & Tjemsland 1996, Mjølnerød 1997, Ueland 1997). Confronting one's own mortality is also reported in other studies to trigger awareness of spirituality (Logan *et al.* 2006, Sørlie *et al.* 2006), and being able to put one's life in God's hands through prayer helps coping (Benedict *et al.* 1994, Heskestad & Tjemsland 1996, Lebel *et al.* 2003, Logan *et al.* 2006). For the participants who could do so, it is worth noting that praying gave confidence in a time of little control. Our study thus provides an integrated theory responding to Penrod's (2001) call for knowledge about how patients use mystical beliefs and practices in managing uncertainty.

The equivalent to our concept of ‘seeking respite’ was found in five other studies. Heskestad and Tjemsland (1996), Fridfinnsdottir (1997) and Logan *et al.* (2006) reported on diverted attention, where patients distracted their minds from thinking of investigations, possible cancer or death. Benedict *et al.* (1994) called this phenomenon diversion, and Lebel *et al.* (2003) named it self-distraction. In acknowledging and respecting patients' individual use of and need for respite, nurses can assist them in regaining strength and enduring uncertainty in the diagnostic phase.

## Conclusion

Our findings are important as they might assist nurses in further developing their role in relation to patients in the diagnostic phase as PWT outlines the complex and dynamic processes patients go through preparing themselves variously for getting a diagnosis. In this process, nurses can be of invaluable support for patients and thereby strengthen their confidence and hope. Further research is needed to further clarify how different patterns of ‘balancing’ influence patients' preparedness for the concluding interview. Further research related to how nurses can assist patients using mostly the ‘balancing’ patterns of rational awaiting and denial is also needed.
